# Identification of Prognostic Related Genes of Tumor Microenvironment Derived From Esophageal Cancer Patients

**DOI:** 10.3389/pore.2021.589662

**Published:** 2021-04-01

**Authors:** Wei Yuan, Jiaqin Yan, Hongtao Liu, Ling Li, BoWen Wu, Can Guo, Mingzhi Zhang

**Affiliations:** ^1^ Department of Oncology, The First Affiliated Hospital of Zhengzhou University, Zhengzhou, China; ^2^ The Academy of Medical Sciences, Zhengzhou University, Zhengzhou, China; ^3^ College of Life Sciences, Zhengzhou University, Zhengzhou, China

**Keywords:** Esophageal cancer, ESTIMATE score, stromal score, immune score, the tumor microenvironment

## Abstract

**Background and Objective:** Esophageal cancer (ESCA) is a commonly occurring cancer worldwide with poor survival and limited therapeutic options. Due to the lack of biomarkers that facilitate early detection, its treatment remains a great challenge. This study aims at identifying the tumor microenvironment (TME)-related genes, which might affect prognosis and accelerate clinical treatment for ESCA patients.

**Methods:** We integrated the expression profiles from ESCA patients in The Cancer Genome Atlas. Then, we determined the stromal and immune scores of each sample using the R package. The Gene Expression Omnibus database was used to validate the expression profile of the key genes.

**Results:** Tumor mutational burden showed a significant difference between the groups of ESCA patients with high and low ESTIMATE scores. We identified 859 intersection genes among patients with different immune and stromal scores. Moreover, gene ontology analysis demonstrated that these 859 intersection genes were closely related to adaptive immune response and regulation of lymphocyte activation. Kyoto Encyclopedia of Genes and Genomes showed the enrichment of cytokine-cytokine receptor interaction and chemokine signaling pathway in the TME. Furthermore, the protein–protein interaction network consisted of 175 nodes. We selected 35 hub genes, including ITGAM, CXCL10, CCR2, CCR5, and CCR1. Of these, 23 intersection genes predicted the overall survival rate. C1QA and FCER1G correlated with overall survival of the ESCA patients in the two databases.

**Conclusion:** We identified a set of stromal and immune score-related prognostic differentially expressed genes that could influence the complexity of the TME. C1QA and FCER1G were identified and validated with respect to their role in the progression of ESCA.

## Introduction

Esophageal cancer (ESCA) is the sixth leading cause of cancer-related death and eighth most common cancer worldwide [1–3]. The two main subtypes of ESCA are esophageal squamous cell carcinoma (ESCC) and esophageal adenocarcinoma (EAC) [4]. Although immunotherapy is a novel therapeutic strategy for ESCA [5, 6], the overall five-year rate of survival remains poor [7, 8]. Therefore, considering the high morbidity and mortality of ESCA, it is essential to identify molecular signatures with prognostic value that affect the tumor microenvironment (TME) in ESCA patients.

The TME of such patients comprises endothelia, fibroblasts, adipocytes, and immune cells and is a key factor for tumor initiation and metastasis [9, 10]. Recent studies have shown that cancer-associated fibroblasts modulate the TME by communicating with tumor and other stromal cells via secretory factors, activating pro-inflammatory signaling, and disrupting immune surveillance [11–13]. Cancer-associated fibroblasts also promote lymph node metastasis in ESCC patients [14]. The TME is involved in all stages of tumorigenesis, i.e., from modulating immune function to promoting angiogenesis and inducing metastasis. Thus, it is crucial to understand how the TME promotes each subtype of ESCA, how specific components in the TME modulate host response to treatment, and what defines and drives the heterogeneity of the TME [15].

This study aims at identifying TME-related genes that affect prognosis and improve clinical treatment of ESCA patients. We used ESTIMATE to describe stromal and immune cells in ESCA from expression data and deduce TME scores, such as stromal score, immune score, and tumor purity. ESTIMATE is an algorithm that can be used to determine the presence of stromal cells and infiltration of immune cells in tumor samples using gene expression matrix data [16]. We integrated the data from expression profiles and overall survival of ESCA from The Cancer Genome Atlas (TCGA) and analyzed the alterations in DNA (base substitutions, indels, gene rearrangements, and copy number variation) and tumor mutational burden (TMB) of each sample using the R Bioconductor package Maftools [17, 18]. Further, we used a bioinformatics assay to elucidate the underlying mechanisms of stromal and immune scores related to differentially expressed genes (DEGs).

## Methods

### Patients and Transcriptional Expression Profiles

Gene expression profiles and mutation data from ESCA patients including 160 tumor samples and 11 normal samples were downloaded from The Cancer Genome Atlas (TCGA) dataset via the GDC data portal (https://portal.gdc.cancer.gov/repository). Clinical data, including age, T stage, N stage, M stage, survival, and histological typing were also obtained from TCGA. One patient whose transcriptomic data and clinical data were not complete was removed. Thus, the TCGA dataset (n = 159) was used as the training set for further analyses. Additionally, the expression profiles from ESCC and EAC patients were downloaded from the Gene Expression Omnibus database (www.ncbi.nlm.nih.gov/geo/), including 34 ESCC samples and 34 normal samples from Series GSE67269-GPL96 and nine normal samples and 12 EAC samples from Series GSE92396.

### Stromal and Immune Scores and Analysis of Prognosis

ESTIMATE is an algorithm that can be used to evaluate the level of immune stromal cell infiltration in cancer tissues using gene expression matrix data. We used the “estimate” package (http://r-forge.r-project.org) to calculate the immune and stromal scores. We divided ESCA patient cohort into two groups based on the medial values of their stromal, immune, and ESTIMATE scores. Kaplan-Meier analysis followed by calculating the *p*-values from the log-rank test was used to compare the difference in survival between low and high groups.

### TMB and Mutant Genome Analysis

TMB was calculated as the total amount of coding errors in somatic genes, base substitutions, insertions, or deletions detected across per million bases. The mutation frequency with number of variants/length of exons (38 million) for each sample was calculated using Perl scripts based on the JAVA8 platform. Mutation data were analyzed using the “maftools” R package that includes multiple analysis modules to perform visualization [18].

### Analysis of DEGs and Functional Pathways

We divided the transcriptome data from the ESCA samples into low and high groups based on the stromal and immune scores using the R software. We used “Limma” to identify the up- and downregulated genes involved with determining the immune and stromal scores in the two groups with |log (fold change)|>1 and false discovery rate<0.05. Heatmaps were generated to represent the DEGs using the “pheatmap” package. Kyoto Encyclopedia of Genes and Genomes (KEGG) and Gene Ontology (GO) pathways were used to analyze the functional role of the intersection genes. GO and KEGG pathway enrichment was analyzed using the “clusterProfiler” package [19]. *p* < 0.05 was considered as statistically significant.

### Protein–Protein Interaction Network and Intersection Genes

PPI networks were constructed using the online STRING tool (https://string-db.org) [20]. All the 859 intersection genes between the immune and stromal score groups were mapped into STRING to determine the correlation among the genes.

### Correlation Between Intersection Genes and Overall Survival

Kaplan-Meier plots were generated to illustrate the correlation between the overall survival of patients and intersection genes using the R package of the survey. Expression of the intersection genes were identified as binary variables (high vs. low) using median expression as the cutoff value for each intersection gene. Odds ratios and values were extracted from the proportional hazards model. *p* < 0.05 was considered statistically significant.

## Results

### Relationships Between High and Low Groups of Stromal, Immune, and ESTIMATE Scores with TMB

**FIGURE 1:**
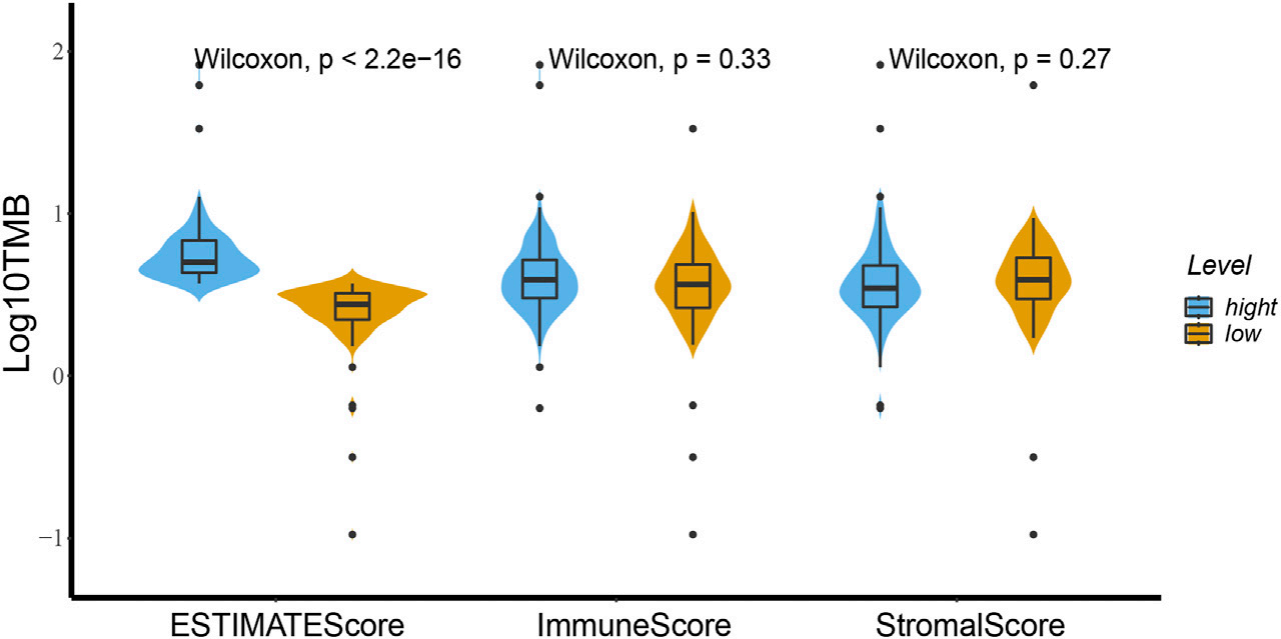
High and low groups of stromal score, immune score and ESTIMATE score relationships with TMB using the Wilcoxon test. There is a distinct difference of TMB between the high and low ESTIMATE score groups for ESCA patients based on the Wilcoxon test (*p* < 2.2e-16). Between the high and low groups of immune score and stromal score the TMB showed no difference (*p* = 0.33 and *p* = 0.27).

As shown in Figure 1, TMB was different in ESCA patients with high and low ESTIMATE scores based on the Wilcoxon test. We divided the patient cohort into high and low-score groups based on the stromal, immune, and ESTIMATE scores followed by analysis of the high and low scores. We found no differences in TMB of the high and low groups based on the stromal and immune scores (*p* = 0.27 and *p* = 0.33, respectively). In contrast, TMB helped distinguish between the high and low ESTIMATE score groups of ESCA patients (*p* = 2.2e-16).

### ESTIMATE and Stromal Scores Correlated with the Clinical Characteristics of ESCA Patients

**FIGURE 2:**
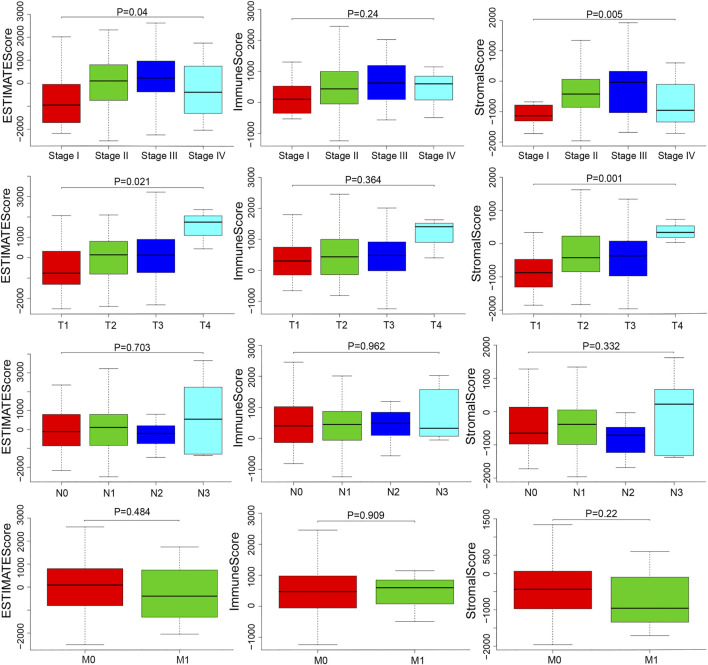
Distributions and comparisons of stromal and immune scores among different ESCA clinical characteristics. ESTIMATE score was correlated with the TNM stage and T stage (*p* = 0.04, *p* = 0.021). Stromal score was correlated with TNM stage and T stage (*p* = 0.005, *p* = 0.001).

**FIGURE 3:**
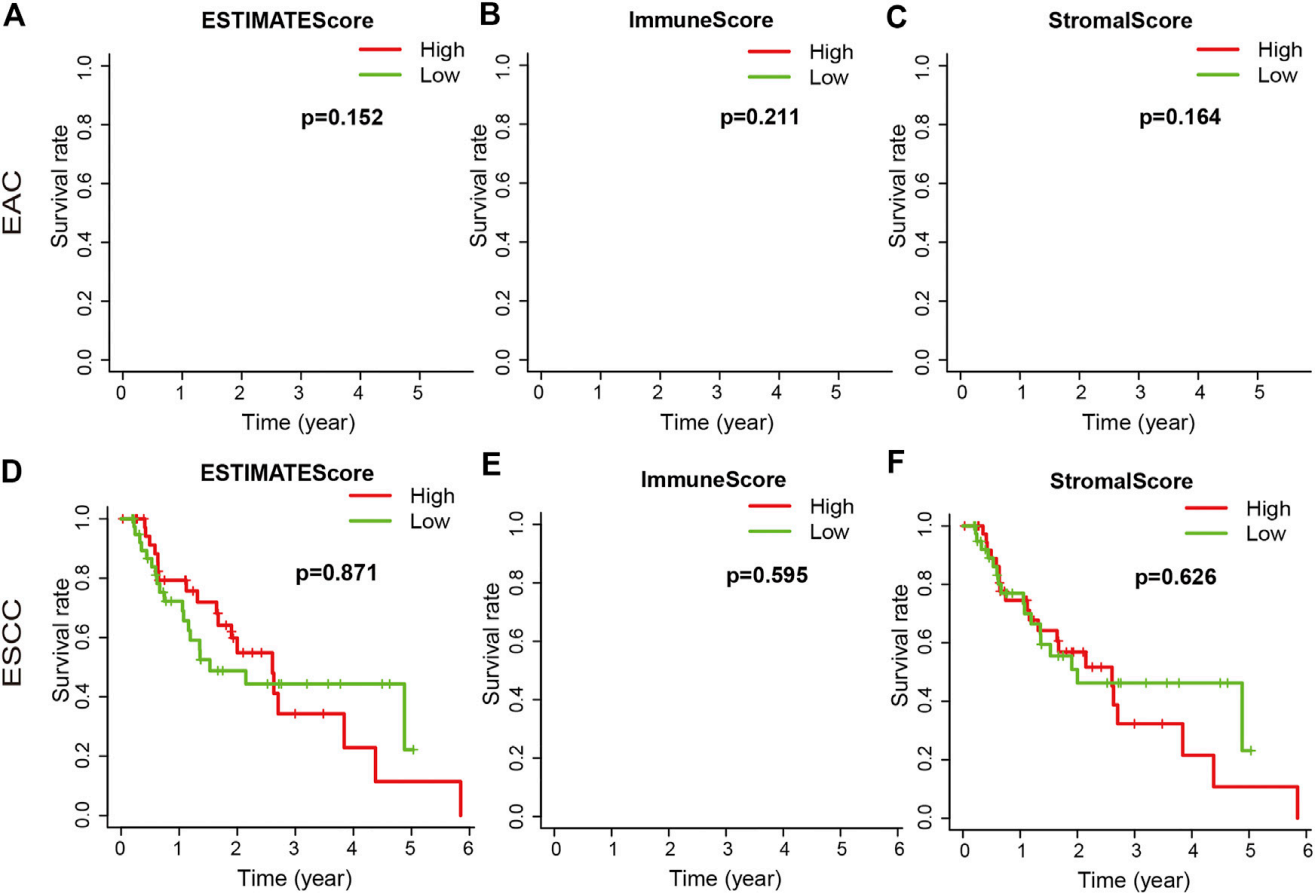
Association of immune, stromal, and ESTIMATE scores with overall survival. The immune, stromal, and ESTIMATE scores of EAC were not associated with overall survival **(A–C)**. The immune, stromal, and ESTIMATE scores of ESCC were not associated with overall survival **(D–F)**.

We downloaded the gene expression profiles and clinical data of 159 ESCA patients with initial pathologic diagnosis from TCGA. The clinical characteristics of patients have been summarized in Table 1. Further analysis showed that ESTIMATE scores correlated with the tumor TNM and T stages (*p* = 0.04 and *p* = 0.021, respectively). Stromal score correlated with tumor TNM and T stages (*p* = 0.005 and *p* = 0.001, respectively). The distribution of immune, stromal, and ESTIMATE scores did not vary with the N and M stages (Figure 2). We divided the ESCA patient cohort into two groups based on their median stromal, immune, and ESTIMATE scores. Subsequently, we used Kaplan-Meier curve analysis to evaluate the correlation between the different scores with overall survival. However, overall survival did not correlate with the immune, stromal, and ESTIMATE scores in the ESCC or EAC patient cohorts (Figure 3).

**TABLE 1:** Clinical characteristics of ESCA patients.

Characteristics		Number of patients	Percentage (%)
Age	< = 60	81	50.94
	>60	78	49.06
Gender	Male	136	14.47
	Female	23	85.53
Histological type	EAC	79	49.69
	ESCC	80	50.31
Vital Status	Alive	96	60.38
	Dead	63	39.62
Status	I	16	10.06
	II	91	57.23
	III	25	15.72
	IV	8	5.03
T	T0	1	0.63
	T1	27	16.98
	T2	37	23.27
	T3	75	47.17
	T4	4	2.52
N	N0	65	40.88
	N1	62	38.99
	N2	9	5.66
	N3	6	3.77
M	M0	119	74.88
	M1	8	5.03
Stromal score	High	1920.26	
	Mean	−468.41	
	Low	−2346.91	
Immune score	High	3388.62	
	Mean	458.34	
	Low	−1242.05	
Estimate score	High	5308.88	
	Mean	−10.07	
	Low	−3419.52	
Radiation therapy	Yes	16	10.06
	No	93	58.49

### Comparison the Gene Expression Profiles with Immune and Stromal Scores in ESCA Patients

**FIGURE 4:**
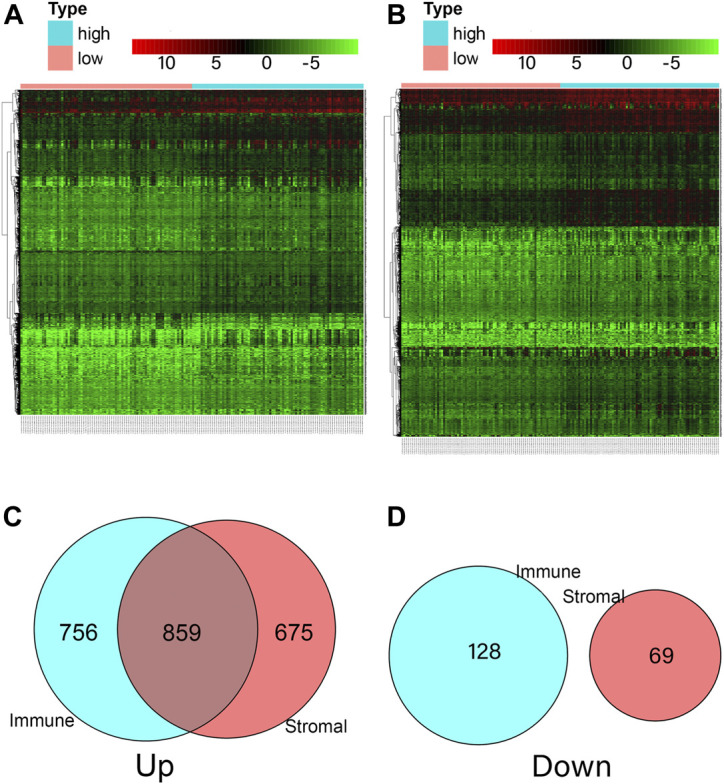
Comparison of ESCA gene expression profile according to immune and stromal scores. **(A)** Heatmap analysis for differential expressions of high immune score and low immune score. **(B)** Heatmap analysis for differential expressions of high stromal score and low stromal score. **(C)** A total of 1,615 immune genes and 1,534 stromal genes were up-regulated, a total of 859 genes were commonly upregulated in the immune and stromal score groups. **(D)** A total of 128 immune genes and 69 stromal genes were down-regulated, and no gene was commonly down-regulated in the immune and stromal score groups.

We analyzed data from transcriptional microarrays of the 159 patients from TCGA to identify the DEGs based on the immune and stromal scores. Comparing the high immune score with low immune scores showed that 1,615 genes were upregulated and 128 genes were downregulated. Comparing stromal scores showed 1,534 upregulated and 69 downregulated genes. A total of 859 DEGs were upregulated in the high-score group, while there were no downregulated genes could be seen in Figure 4 (https://doi.org/10.5281/zenodo.4270637).

### KEGG Pathways and GO Biological Enrichment Analyses

**FIGURE 5:**
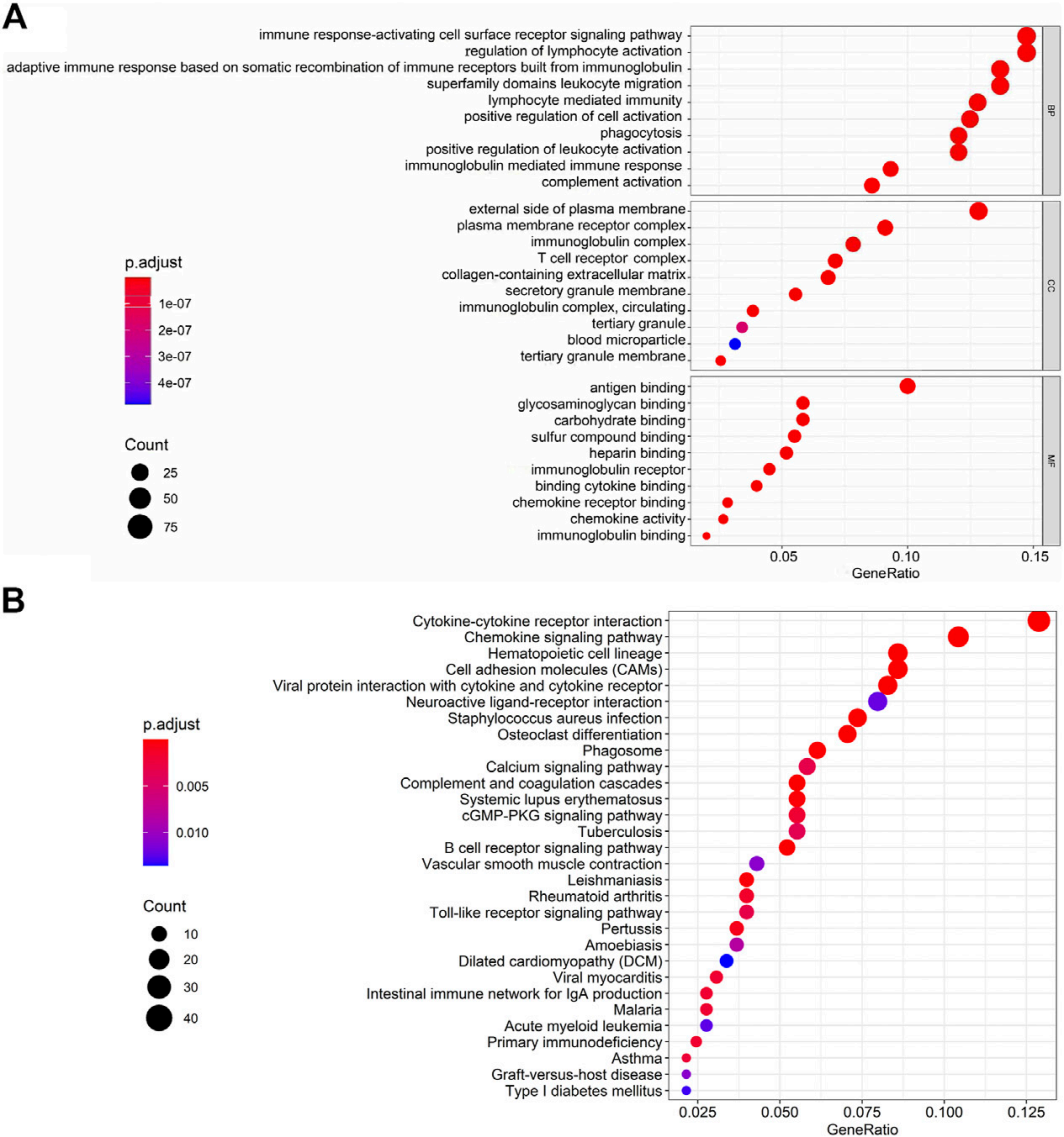
GO terms and KEGG interpretation for functions of intersection genes in ESCA. **(A)** GO biological processes including BP, CC and MF. **(B)** The KEGG pathways were mainly enriched for Cytokine-cytokine receptor interaction.

We represented the top 10 GO biological processes, cellular component, and molecular functions. We found 791 GO-associated terms for 665 genes, that were closely related to the immune response-activating cell surface receptor signaling pathway, external side of the plasma membrane, and antigen binding as the biological process, cellular component, and molecular function, respectively. The pathways associated with cytokine-cytokine receptor interaction was the primarily enriched KEGG pathway (Figure 5 and Supplementary Tables S1, S2).

### PPI Network Construction and Modules Selection

**FIGURE 6:**
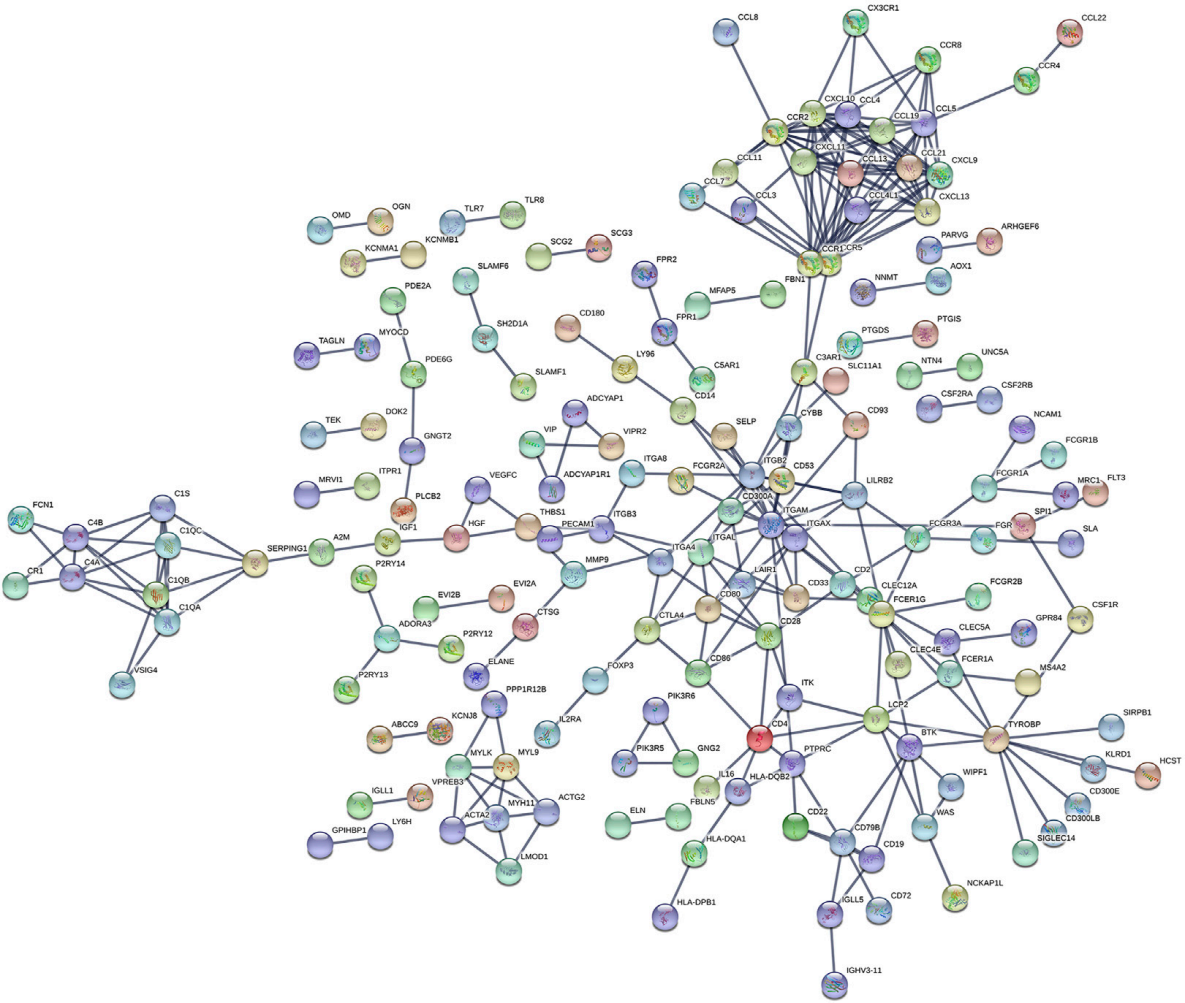
The PPI network of intersection genes between the immune and stromal score groups. The PPI network of DEGs was constructed by 175 nodes and 295 edges, including 859 upregulated genes.

**FIGURE 7:**
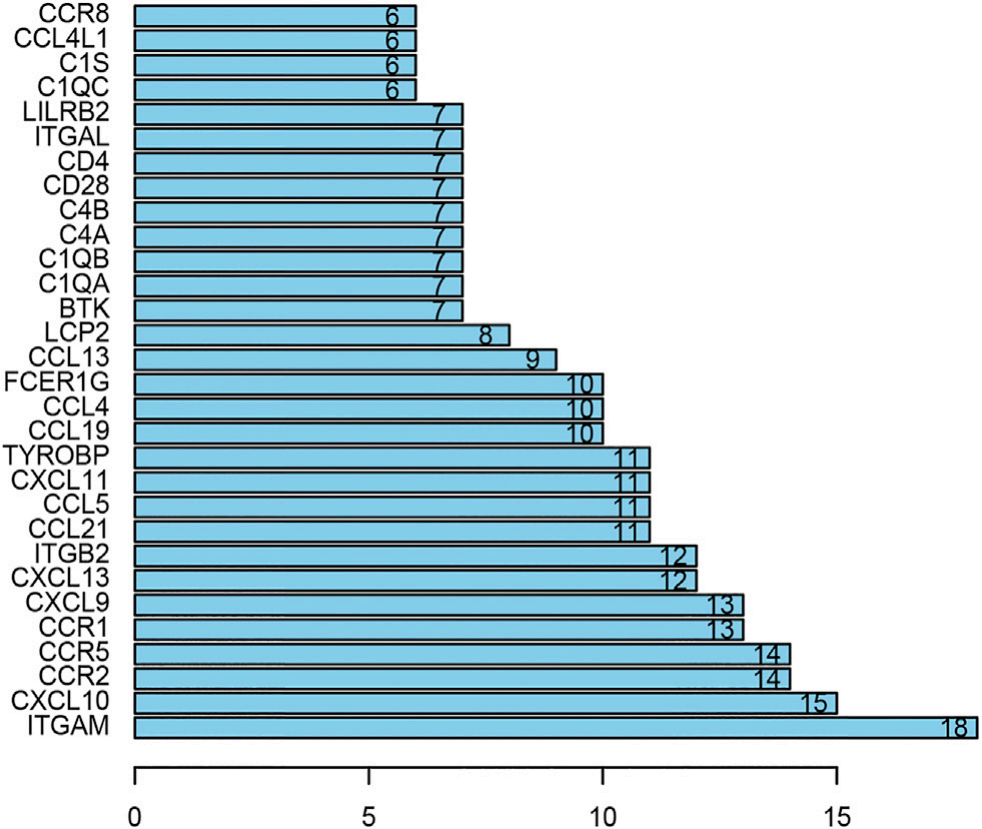
The hub genes in the PPI network. There are 35 genes selected as hub genes, such as ITGAM, CXCL10, CCR2, CCR5 and CCR1, enriched in a module when degrees c ≥ 6 were set as the cutoff criterion.

The PPI network including DEGs was constructed using 175 nodes and 295 edges, including 859 upregulated genes, using the STRING online tool as shown in Figure 6 (https://doi.org/10.5281/zenodo.4270637). We selected 35 hub genes, such as ITGAM, CXCL10, CCR2, CCR5, and CCR1, that were enriched in the module using degrees c ≥ 6 as the cutoff criterion. The connected nodes for each intersection gene have been shown in Figure 7.

### Survival Analysis of Intersection Genes

**FIGURE 8:**
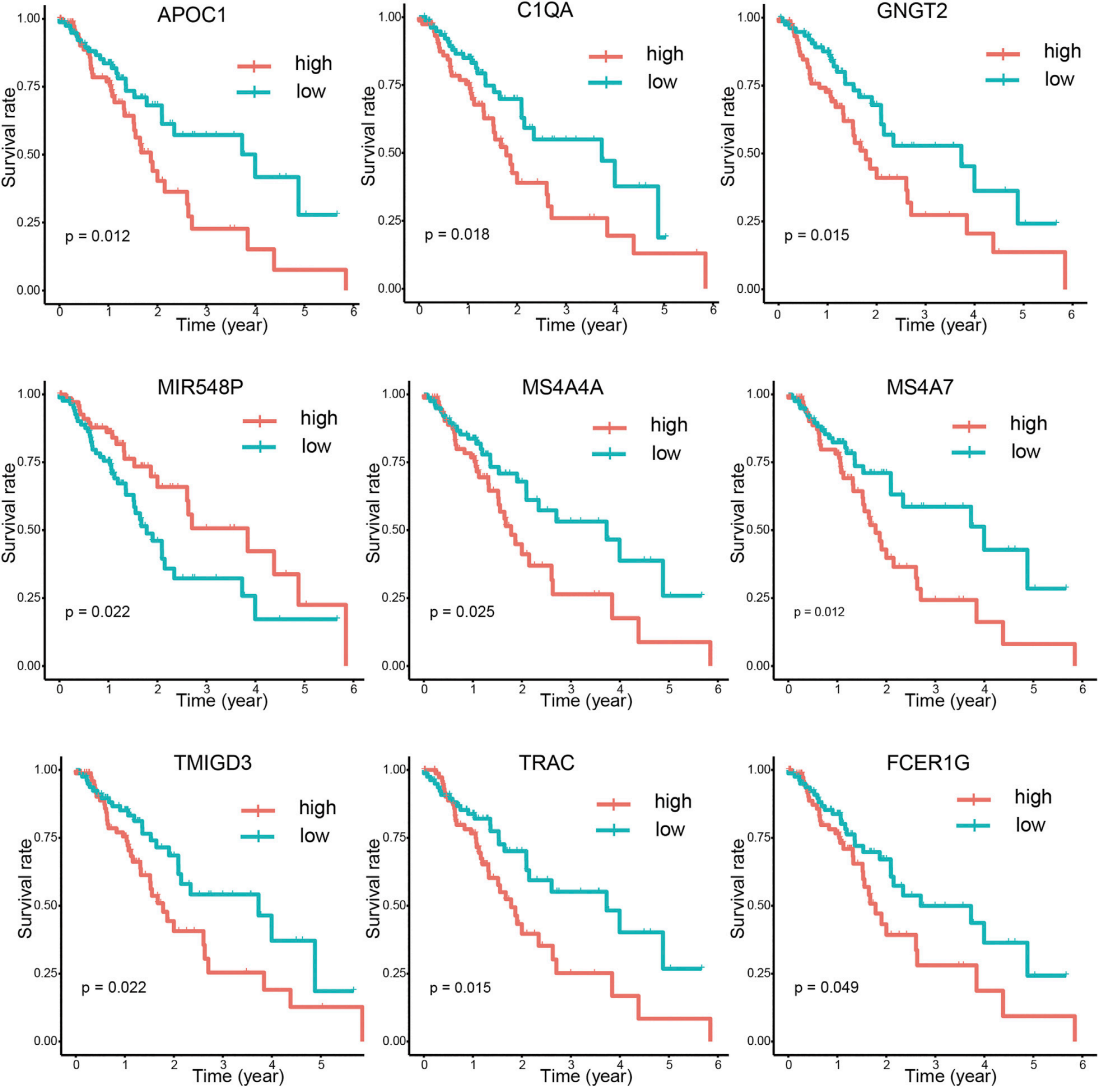
Correlations between DEG expression and overall survival. The survival curves of selected DEGs from the high (red line) and low (blue line) gene expression groups were generated using the weighted Kaplan-Meier analysis method (*p* < 0.05 in the log-rank test).

**FIGURE 9:**
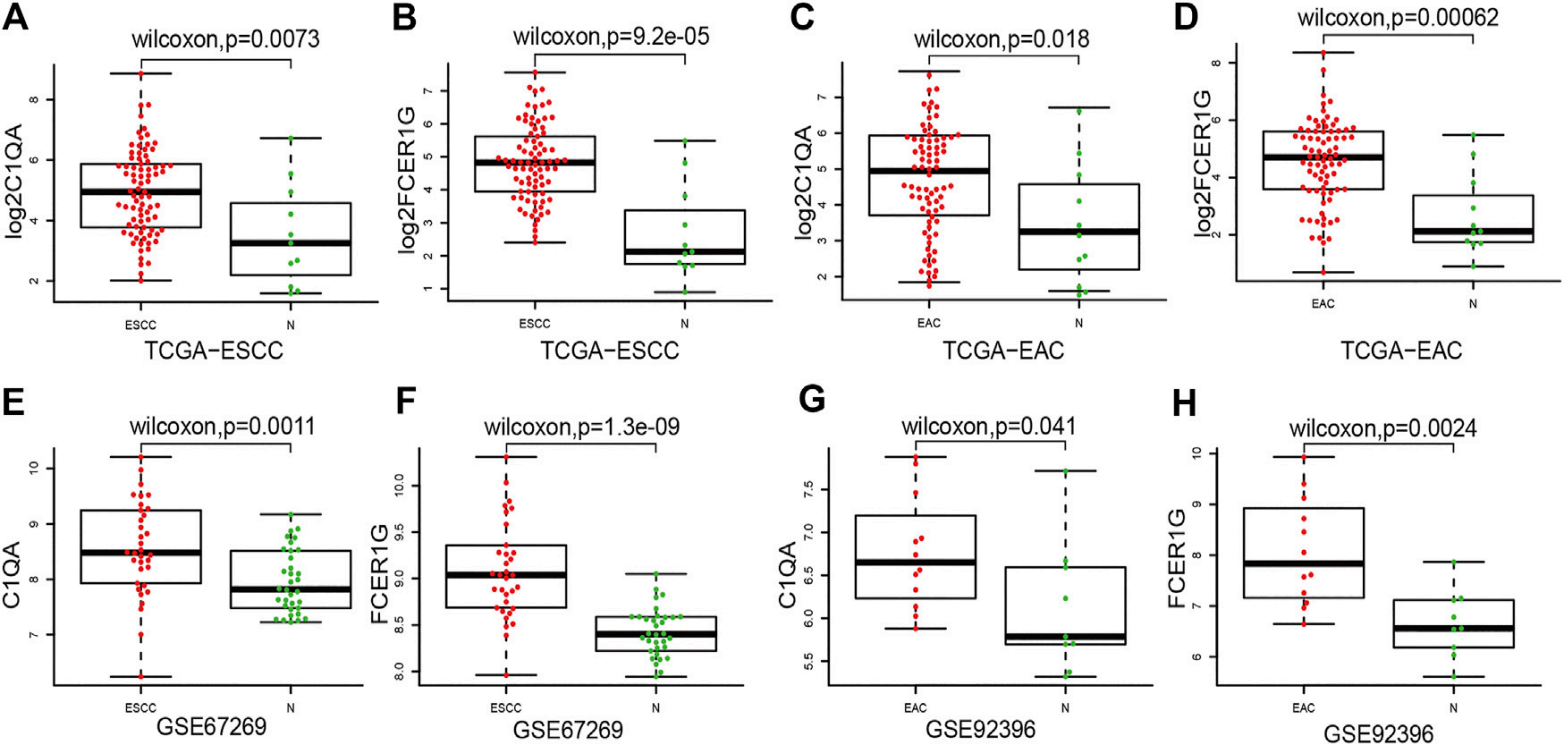
Compared ESCC and EAC tissues with normal tissues for C1QA and FCER1G mRNA expressions. **(A–D)** Expression of C1QA and FCER1G mRNA in TCGA database **(E–F)** Expression of C1QA and FCER1G mRNA in ESCC-GSE67269. **(G–H)** Expression of C1QA and FCER1G mRNA in EAC-GSE92396.

After integrating the mRNA expression profile of the intersection genes and clinical information, Kaplan-Meier survival curves were obtained using the TCGA cohort. We found that 23 of the 859 intersection genes predicted overall survival rate (*p* < 0.05, Supplementary Tables S3). The Kaplan-Meier survival curve illustrated the effects of nine main genes on the overall survival of ESCA patients (Figure 8). C1QA and FCER1G comprised the nodes of the intersection genes that helped construct the PPI network; these were identified to be prognostic genes. The mRNA levels of C1QA and FCER1G were elevated in ESCC and EAC tissues as compared to those in normal tissues (Figure 9).

## Discussion

Despite advancements in diagnostics and therapeutics, the prognosis of ESCA remains poor [2–4]. There is accumulating evidence that a comprehensive understanding of the molecular composition of ESCA requires attention to not only tumor cells but also the TME, which contains diverse cell populations, such as stromal and immune cells, that interact with tumor cells and participate in all stages of tumorigenesis [15]. In this study, we used ESTIMATE to determine the role of stromal and immune cells using the expression data from ESCA patients and calculate stromal score, immunity score, and tumor purity. We identified the stromal and immune scores associated with prognostic DEGs, and developed 23 stromal and immune score-based gene signatures as a prognostic stratification tool for ESCA patients.

TMB is emerging as a potential biomarker for cancer; high TMB is beneficial with immune checkpoint blockade therapy [21]. High TMB inhibits immune cell infiltration and promotes anti-cancer inflammatory response [22]. In this study, TMB was significantly different in the high and low ESTIMATE score groups of ESCA patients. The result that may be due to tumor purity and immune infiltration is controversial. Thus, our future studies will focus on determining the correlation between TMB and ESTIMATE scores. Stromal score correlated with tumor TNM stage in this study. Immune scores correlate with the prognostic ability of the current TNM stage [23]. The immune microenvironment is a potential therapeutic target and immune checkpoint inhibitor. Although there was no statistical difference between these scores and overall survival in this study, a high stromal score predicts poor prognosis and high pathological stage in primary gastric cancer; moreover, immune scores were associated with better overall survival in patients with cervical squamous cell carcinoma [24, 25].

We identified 859 upregulated intersection genes between patients with varying immune and stromal scores. These 859 intersection genes were associated with biological processes involving the TME, including adaptive immune response, immune response-activating cell surface receptor signaling pathway, and lymphocyte-mediated immunity. These processes inhibit tumor progression and metastasis, thereby improving patient survival. The molecular functions of intersection genes were related to antigen binding, immunoglobulin receptor binding, and heparin binding. However, the function of the 859 intersection genes requires further investigation. As illustrated in the PPI network, 35 genes were selected as hub genes, such as ITGAM, CIQA, FCER1G, CXCL10, CCR2 and CCR5. C1QA and FCER1G also predicted the rate of overall survival.

C1QA encodes the A-chain polypeptide of serum complement subcomponent C1q, which is associated with C1r and C1s, to yield the first component of the serum complement system [26]. C1q deficiency is associated with lupus erythematosus and glomerulonephritis [27]. A polymorphism associated with C1qA decreases complement activity, thereby reducing the hematogenous spread of breast cancer [28, 29]. Non-bone marrow-derived C1q helps prevent tumor progression by facilitating cancer cell seeding and promoting angiogenesis [30]. Immune-related factors, including C1QA, play an important role in the development of EAC, suggesting the potential of these factors as therapeutic targets for EAC [31].

The high affinity Ig E receptor, FCER1G, is a key molecule involved in allergic reactions. This tetramer is composed of one alpha, one beta, and two gamma chains [32]. Gamma chains are also subunits of other Fc receptors [32]. FCER1G is essential for chronic inflammation and plays an important role in death-activating signaling, inducing apoptosis [33]. FCER1G levels negatively correlate with the progression of multiple myeloma. FCER1G serves as a hub gene involved in the development of lung adenocarcinoma [34]. Further, FCER1G is crucial in the prognosis of prostate cancer [35].

This study identified 23 prognostic genes, including MS4A7, APOC1, TRAC, GNGT2, C1QA, NKX6-1, TMIGD3, MIR548P, MS4A4A, TRAV16, AF127936.1, FCGR3A, ALOX5AP, TRAV12-2, MS4A6A, TRAV6, EVI2A, TRBV4-1, SPOCK2, FCER1G, TRAV8-4, ARHGEF6, and CD300A. Among these, C1QA and FCER1G comprised the nodes of the intersections in the PPI network. Thus, C1QA and FCER1G were used to validate the progression of ESCA. These data also provide a foundation for further studies on the correlation between the TME and C1QA and FCER1G expression. This study highlights the utility of the components of the TME as therapeutic targets; however, future studies should focus on determining the potential of the TME in predicting the prognosis of ESCA patients.

## Conclusion

In summary, this study identified a set of stromal and immune scores related to the prognostic DEGs using the ESTIMATE algorithm. C1QA and FCER1G were the hub genes that were validated for their role in the progression of ESCA that could help understand the complexity of the TME. However, this study has some limitations. First, we analyzed a relatively small cohort. Owing to the lack of sufficient data from the databases, many potential DEGs remain uninvestigated. Second, since the TME has different roles during tumor progression and metastasis, the findings of this study do not provide a holistic picture of the immune and stromal scoring system across the different stages of ESCA. Nevertheless, the study findings demonstrate the clinical significance and therapeutic potential for ESCA. Future studies should employ well-designed prospective clinical trials to highlight the role of the TME in tumor progression and metastasis.

## Data Availability

Publicly available datasets were analyzed in this study. This data can be found here: <b>https://doi.org/10.5281/zenodo.4050666 and https://github.com/zzyuanwei/Identification-of-prognostic-related-genes-of-TME-derived-from-ESCA</b>.

## Abbreviations

ESCA, esophageal cancer; TME, tumor microenvironment; TCGA, the cancer genome atlas; TMB, tumor mutational burden; GO, gene ontology; KEGG, kyoto encyclopedia of genes and genomes; PPI, protein–protein interaction; DEGs, differential expression genes; ESCC, esophageal squamous cell carcinoma; EAC, esophageal adenocarcinoma.
