# Supplementary material for: Identification of Prognostic Related Genes of Tumor Microenvironment Derived From Esophageal Cancer Patients
Source: Pathol Oncol Res. 2021 Apr 1;27:589662. doi: 10.3389/pore.2021.589662 (PMC8262216; doi:10.3389/pore.2021.589662)
Supplement: Supplementary file 2 [file DataSheet2.ZIP › Table/Tab1.docx]

Clinical characteristics of ESCA patients in TCGA.

| Characteristics |  | **Number of patients** | | Percentage (**%)** | |
| --- | --- | --- | --- | --- | --- |
|  |  | 81  78 |  | |  |
| Age | <=60  >60 |  | 50.94  49.06 | |  |
| Gender | Male  Female | 136  23 | 14.47  85.53 | |  |
| Histological type | EAC  ESCC | 79  80 | 49.69  50.31 | |  |
| Vital status | Alive  Dead | 96  63 | 60.38  39.62 | |  |
| Stage | I  II  III  IV | 16  91  25  8 | 10.06  57.23  15.72  5.03 | |  |
| T | T0  T1  T2  T3  T4 | 1  27  37  75  4 | 0.63  16.98  23.27  47.17  2.52 | |  |
| N | N0  N1  N2  N3 | 65  62  9  6 | 40.88  38.99  5.66  3.77 | |  |
| M | M0  M1 | 119  8 | 74.84  5.03 | |  |
| Stromal Score | High  Mean  Low | 1920.26  -468.41  -2346.91 |  | |  |
| Immune Score | High  Mean  Low | 3388.62  458.34  -1242.05 |  | |  |
| ESTIMATE Score | High  Mean  Low | 5308.88  -10.07  -3419.52 |  | |  |
| Radiation therapy | Yes  No | 16  93 | 10.06  58.49 | |  |
|  |  |  |  | |  |

The entire TCGA data (n=159) was as the training set for further analyses. The

incomplete clinical information is not displayed in the table.
