# Supplementary material for: Identification of Prognostic Related Genes of Tumor Microenvironment Derived From Esophageal Cancer Patients
Source: Pathol Oncol Res. 2021 Apr 1;27:589662. doi: 10.3389/pore.2021.589662 (PMC8262216; doi:10.3389/pore.2021.589662)
Supplement: Supplementary file 2 [file DataSheet2.ZIP › Table/Tab1.pdf]

Clinical characteristics of ESCA patients in TCGA.

Characteristics		Number of patients	Percentage (%)
Age	<=60	81	50.94
	>60	78	49.06
Gender	Male	136	14.47
	Female	23	85.53
Histological type	EAC	79	49.69
	ESCC	80	50.31
Vital status	Alive	96	60.38
	Dead	63	39.62
Stage	I	16	10.06
	II	91	57.23
	III	25	15.72
	IV	8	5.03
T	T0	1	0.63
	T1	27	16.98
	T2	37	23.27
	T3	75	47.17
	T4	4	2.52
N	N0	65	40.88
	N1	62	38.99
	N2	9	5.66
	N3	6	3.77
M	M0	119	74.84
	M1	8	5.03
Stromal Score	High	1920.26	
	Mean	-468.41	
	Low	-2346.91	
Immune Score	High	3388.62	
	Mean	458.34	
	Low	-1242.05	
ESTIMATE Score	High	5308.88	
	Mean	-10.07	
	Low	-3419.52	
Radiation therapy	Yes	16	10.06
	No	93	58.49

The entire TCGA data (n=159) was as the training set for further analyses. The incomplete clinical information is not displayed in the table.
